# EEG and Eye Tracking Signatures of Target Encoding during Structured Visual Search

**DOI:** 10.3389/fnhum.2017.00264

**Published:** 2017-05-16

**Authors:** Anne-Marie Brouwer, Maarten A. Hogervorst, Bob Oudejans, Anthony J. Ries, Jonathan Touryan

**Affiliations:** ^1^Perceptual and Cognitive Systems, Netherlands Organisation for Applied Scientific Research (TNO)Soesterberg, Netherlands; ^2^U.S. Army Research LaboratoryAberdeen, MD, United States

**Keywords:** EEG, pupil size, fixation, SRP, FRP, visual search, target detection, BCI

## Abstract

EEG and eye tracking variables are potential sources of information about the underlying processes of target detection and storage during visual search. Fixation duration, pupil size and event related potentials (ERPs) locked to the onset of fixation or saccade (saccade-related potentials, SRPs) have been reported to differ dependent on whether a target or a non-target is currently fixated. Here we focus on the question of whether these variables also differ between targets that are subsequently reported (hits) and targets that are not (misses). Observers were asked to scan 15 locations that were consecutively highlighted for 1 s in pseudo-random order. Highlighted locations displayed either a target or a non-target stimulus with two, three or four targets per trial. After scanning, participants indicated which locations had displayed a target. To induce memory encoding failures, participants concurrently performed an aurally presented math task (high load condition). In a low load condition, participants ignored the math task. As expected, more targets were missed in the high compared with the low load condition. For both conditions, eye tracking features distinguished better between hits and misses than between targets and non-targets (with larger pupil size and shorter fixations for missed compared with correctly encoded targets). In contrast, SRP features distinguished better between targets and non-targets than between hits and misses (with average SRPs showing larger P300 waveforms for targets than for non-targets). Single trial classification results were consistent with these averages. This work suggests complementary contributions of eye and EEG measures in potential applications to support search and detect tasks. SRPs may be useful to monitor what objects are relevant to an observer, and eye variables may indicate whether the observer should be reminded of them later.

## Introduction

Visual search is a common task that is performed when looking for a singing bird in the trees, when checking a poster for graphical errors or when searching an environment for suspicious objects. We are interested in EEG and eye variables as potential sources of information about the underlying processes of target detection and encoding during visual search, i.e., a task where the eyes move. As elaborately discussed in “Application” Section, monitoring target detection and encoding on the basis of implicit variables could be useful in a number of applications.

### Brain and Eye Correlates of Target Detection

The literature describes several EEG and eye indicators of target detection. Observers usually fixate longer on targets than non-targets in a search task (e.g., Brouwer et al., 2013; Jangraw et al., 2014; Wenzel et al., 2016). Also, several studies showed stronger pupil dilation responses to target compared to non-target stimuli (Nieuwenhuis et al., 2011; Hong et al., 2014). Finally, the P300 event related potential (ERP), a positive peak occurring in the EEG signal roughly 300 ms after a sensory event, indicates that an observer’s attention has been drawn. It has been shown that the P300 reliably distinguishes between top-down defined “targets” and “non-targets”, e.g., in cases where observers are asked to pay attention to the letter “p” presented in a sequence of successively flashed letters (e.g., Farwell and Donchin, 1988).

While in studies on pupil dilation and P300 participants usually kept their eyes still, some studies showed similar findings when participants were actively and purposefully moving their eyes. Jangraw et al. (2014) found that, for a realistic visual search scenario, pupil size locked to target fixation onset increased relative to non-target fixation. There is a growing body of research on ERPs that are not time locked to stimulus onset as determined within an experimental paradigm, but to ocular-based events such as saccades and fixations (saccade-related potentials, SRPs or fixation-related potentials, FRPs respectively) as a means to determine whether observers are looking at a target (i.e., a top-down defined relevant object). For some of the early studies (e.g., Hale et al., 2008; Luo et al., 2009), differences found between target and non-target SRPs could have been due to confounding factors such as systematic target vs. non-target differences in saccade length, low level visual features, or motor preparation to press a button. However, by now it is clear that ERPs following fixation of a target are different than ERPs following fixation of a non-target and that these differences are associated with top-down stimulus processing (e.g., Dandekar et al., 2012a,b; Kamienkowski et al., 2012; Brouwer et al., 2013).

### Brain and Eye Correlates of Missed Targets

In the current study, we examine fixation duration, pupil size and SRPs in a structured visual search task. We are especially interested in encoding failures, i.e., failing to report a fixated target. Observers can fail to report fixated targets for different reasons. They may have “really” missed the targets, e.g., because the type of target was difficult to identify (perceptual identification failures). Another possible reason is that after target identification, observers forgot the target before it was time to report, for example, because they were involved in another task (memory encoding failures).

In the case that observers did not identify the target, we expect eye and SRP features for misses to be similar to non-targets—observers simply did not perceive the target as being a target. This hypothesis is supported by research by Dias et al. (2013) who studied SRPs following fixations of not-reported targets under circumstances that misses were likely due to not identifying the target. In their task, participants searched a display filled with rectangular objects where the target was defined by a combination of visual features that changed every trial. For instance, a target could be a vertical bar consisting of a red bar on the left and a yellow one on the right that was presented between non-targets that also consisted of vertical colored bar combinations. After finding the target, participants had to immediately report finding it. The average miss SRP could not be distinguished from the average non-target SRP, while the average hit SRP stood out and was consistent with a P300. Dias et al. (2013) found that misses were associated with relatively high EEG alpha activity which has also been linked to lapses of attention (e.g., Vázquez Marrufo et al., 2001).

In the case that targets are identified but not reported due to a memory encoding failure, we do not expect features for misses to be the same as for non-targets. Here, misses are detected as targets at the time of fixation. However, to the extent that differences in target vs. non-target SRPs reflect differences in allocated attention, where this difference matters for storage in memory, SRP waveforms following these missed targets could fall in between target and non-target SRPs. Evidence that is partly in line with this expectation has been found in previous P300 studies. In these studies, participants were asked to remember as many words as possible of a list of sequentially presented words. P300s following presentation of words that were later remembered were compared to those that were later forgotten. Remembered words tended to correspond with larger P300s. However, these effects were small and interacted with primacy and recency effects as well as the type of rehearsal or encoding strategy of the participants (Karis et al., 1984; Fabiani et al., 1990; Azizian and Polich, 2007; Kamp et al., 2012). For instance, Azizian and Polich found larger P300 amplitudes for recalled compared to forgotten words only for words at the beginning of the list, and Fabiani et al. (1990) only found a positive relation between P300 and recall when participants used a rote strategy (repeating the words to themselves) for remembering. As of yet, we do not know whether there is a relation between the P300 and later recall of targets in a visual search task.

Fixation duration may reflect or may enable more attention and deeper processing of the fixated object. Therefore, we not only expect fixation duration to be longer for targets than for non-targets, but also for hits compared to misses.

We discussed that SRPs and fixation duration as signatures for missed targets may be in between hit target and non-target values. The same may hold for pupil size, i.e., hit targets may be associated with larger pupil size than missed targets. However, the reverse may be found as well. In case of a search task where targets are likely to be forgotten because the observer is simultaneously attending to another task, we expect momentary high workload to be associated with misses. Since workload or memory load is strongly related to pupil size (Kahneman and Beatty, 1966; Beatty, 1982; Hogervorst et al., 2014), also specifically with memory load during visual search (Porter et al., 2007), we expect it to be larger for misses than for hits in our task.

### Current Study

In the current study we examine the general association between SRP and eye features on the one hand and whether a fixated object is a target and is going to be missed on the other hand. In addition, we examine how well we can distinguish between targets and non-targets, and between hits and misses on a single fixation basis and which combination of these sources of information gives us the best result. A surge of recent studies show that it is possible to distinguish between target and non-target SRPs above chance on a single SRP basis, also in rather challenging circumstances. For instance, Ušćumlić and Blankertz (2016) show a single trial distinction when moving stimuli are involved; single trial classification has been shown for a mixture of foveally and parafoveally identified stimuli (Brouwer et al., 2014; Wenzel et al., 2016); and it has been shown when using more natural stimuli such as looking for a face in a crowd (Kaunitz et al., 2014) or when viewing signs during navigating a virtual environment (Jangraw et al., 2014). Some previous studies have shown that combining SRP features and eye related features increased classification performance for targets and non-targets (Jangraw et al., 2014; Wenzel et al., 2016). Thus, for target and non-target distinction, eye and brain signals can potentially add complimentary information. It remains to be seen how this works out for the distinction between hits and misses.

In our task, participants performed a structured visual search task consisting of scanning 15 locations on a screen. Target locations were reported after scanning all locations. An auditory math task was performed in the high load condition but ignored in the low load condition. In pilot experiments we verified that performing such a double task results in failures to report targets. With respect to the SRP, we expect a larger P300 for targets compared to non-targets, and possibly a higher P300 for hit compared to missed targets. We expect longer fixation duration for targets compared to non-targets, and longer fixation duration for hits than for misses. Pupil size may be larger for targets than for non-targets, and—through a general association between high workload and pupil size—larger for misses than for hits.

## Materials and Methods

### Participants

Twenty-one participants (nine males, 12 females) between the age of 19 and 30 years (average age: 23) were recruited through the participant pool of the Netherlands Organization for Applied Scientific Research (TNO). None of the participants wore glasses. Each participant received a monetary reward for his or her time and travel costs. This study was carried out in accordance with the recommendations of the Human Research Protections Official (HRPO) and the TNO Institutional Review Board (TCPE) with written informed consent from all subjects. All participants signed an informed consent form in accordance with the Declaration of Helsinki. This study was approved by the HRPO and TCPE and conducted in accordance with the Army Research Laboratory’s IRB requirements (32 CFR 219 and DoDI 3216.02).

### Materials

The task was presented on a 19″ flat-screen monitor (Dell 1907FP Flatpanel 19″, display size 37.5 × 30 cm). The screen resolution was 1280 × 1024 and the refresh rate was set at 60 Hz. Participants’ eyes were located approximately 40 cm from the screen. Audio output was coming from a dual speaker set (TEAC PowerMax 60/2) placed left and right of the screen.

Gaze and pupil size were recorded at 60 frames per second using SmartEyePro V6.1.6 (Smart Eye AB, Göteburg, Sweden). This system consists of two cameras (Basler acA640-120 gm, HR 8.0 mm lens) placed at the left and right side of the screen.

EEG and EOG signals were recorded using an ActiveTwoMK II system (BioSemi, Amsterdam, Netherlands) with a sampling frequency of 512 Hz. For EEG, 32 active silver-chloride EEG electrodes were placed according to the 10–20 system and were referenced to the Common Mode Sense (CMS) active electrode and Driven Right Leg (DRL) passive electrode. Four EOG electrodes (BioSemi Flat Active electrodes, Amsterdam, Netherlands) were used to record eye movement. Two EOG electrodes were placed at the approximately 0.5 cm off the lateral canthi of both eyes, and were used to record horizontal eye movement. Another two EOG electrodes were placed above and below the left eye to record vertical eye movement and blinks. The electrode offset of all electrodes was below 25.

### Task and Design

The experiment featured two tasks: a monitoring task and an auditory math task. In the high load condition, participants performed both tasks. In the low load condition, they only needed to perform the monitoring task, even though the math task was still played to keep auditory stimulation constant across conditions.

#### Monitoring Task

**Figure 1:**
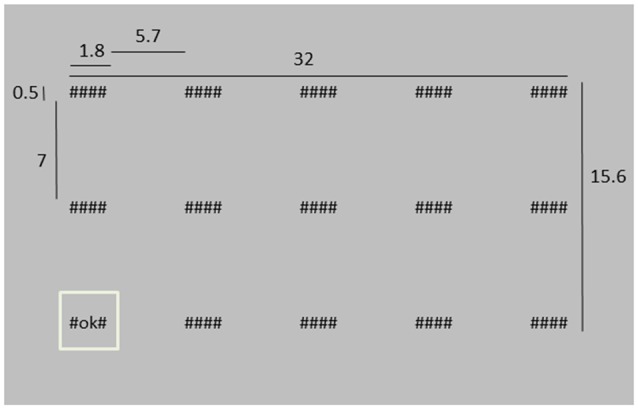
**Depiction of the stimulus screen at a moment where the lower left system is highlighted**. Dimensions are indicated in cm. The distance between the screen and the eyes was approximately 40 cm, i.e., in degrees of visual angle, the “####” system was about 2.6 × 0.7° of visual angle.

Participants were asked to monitor 15 “systems”, represented by strings of symbols on a screen and placed in three rows of five columns. There were three different system conditions: hidden (“####”), working as intended (“#OK#”) or system failure (“#FA#”). At the start of a trial, all system conditions were hidden. Then, each of the systems was successively highlighted for 1 s (1027 ms) by displaying a square around it while its condition changed from “####” into either “#OK#” or “#FA#” (Figure 1 shows an example of the stimulus display, and also presents the dimensions of the different stimulus elements). Highlighting the systems happened in random order, except for that two subsequently presented systems were never further apart than two steps in horizontal direction and one in vertical direction, or two vertical and one horizontal. The next highlighted system was in peripheral vision, such that we could not distinguish between “#OK#” or “#FA#” without making a saccade. After all system conditions had been shown, empty boxes appeared at the system locations and the participant had to indicate which systems failed during the trial by clicking the appropriate boxes with the left mouse button. When finished, the participant pressed an OK button at the top left of the screen. Every trial, two, three or four “#FA#”s were presented. The amount (two, three or four) and the “#FA#” locations were chosen randomly.

#### Math Task

The math task was an aurally presented sum consisting of six numbers between 6 and 12. Only addition (+) and subtraction (−) operations were used. An example is “8−6 + 10−12 + 11 + 7”. The first number was presented 1 s after the start of the monitor task, and every 2660 ms another number was presented. Thus, the last number was presented after 14.3 s. Performing this task involves attention and working memory. When participants had to perform the math task (i.e., in the high load condition), they were required to give the answer of the sum after having indicated where the “#FA#”s were located. This was done by typing the answer and pressing enter. In order to motivate participants to perform the math task, they received feedback on their answer. If the answer was incorrect, the correct answer was shown.

For each of the load conditions, participants performed eight blocks of 11 trials. High and low load conditions were presented alternately, starting with the high load condition.

### Procedure

After a general explanation and signing the informed consent, the EEG and EOG electrodes were attached. During this time, the participant had time to read detailed instructions. Participants were asked to take a comfortable position in front of the screen. Even though they were able to move freely, they were instructed to minimize their head and body movements. Before the task began, a four point-calibration was performed to calibrate the SmartEye system. There was a few minutes break after eight blocks of trials, i.e., half-way through the experiment. The participants indicated when they were ready to continue.

### Analysis

#### Electrophysiological Data

EEG and EOG data was resampled to 256 Hz. Bad EEG channels were identified as channels with standard deviations exceeding five times the median standard deviation over all channels, after bandpass filtering between 0.5 and 32 Hz. This affected 1–3 channels for five participants. The unfiltered data from bad channels were replaced by the weighted average of unfiltered data from the surrounding channels. Next, the EEG-data was re-referenced to the mean of all unfiltered data excluding the bad channels. The resulting signals were submitted to bandpass filtering between 0.5 and 32 Hz.

**Figure 2:**
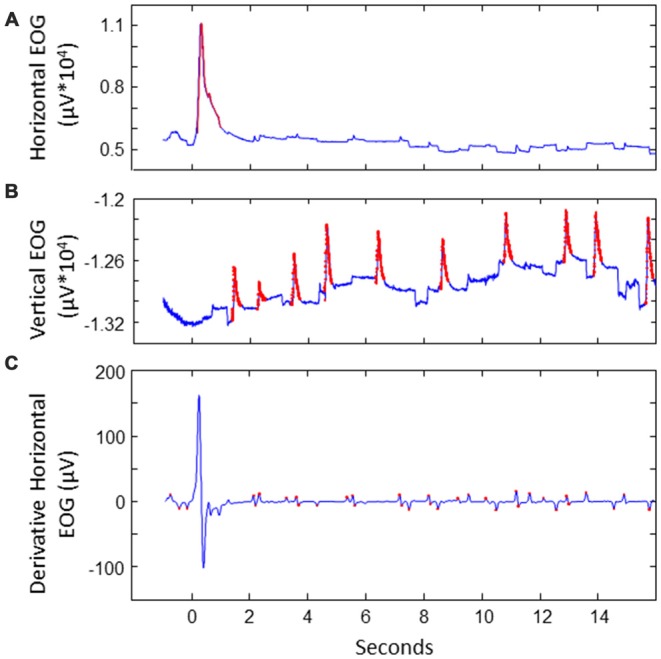
**Illustration of extracting saccades of interest for a sample trial**. **(A)** Detecting noise (indicated in red) in EOG. This example shows the horizontal EOG. Noise was cut out and interpolated. **(B)** Detecting blinks in vertical EOG. Blinks were cut out and interpolated. **(C)** Detecting potential saccades of interest in the derivative of the cleaned EOG. This example shows the horizontal EOG.

We extracted saccades to divide the data in saccade-locked segments. This was done as follows (see also Figure 2). First, horizontal and vertical EOG were cleaned from noise. This was done by detecting values that exceeded five times the standard deviation. The signal around these peaks was cut out and interpolated. Next, blinks were detected and removed in vertical EOG: after band pass filtering between 2 Hz and 100 Hz, peaks exceeding a threshold of three times the standard deviation were considered to be blinks, removed and interpolated. Derivatives of the vertical and horizontal cleaned EOG signals were calculated using a derivative Gaussian filter with a standard deviation (sigma) of eight samples (about 31 ms). Values exceeding four times the standard deviation were associated with potential stimulus-to-stimulus saccades. We then looked at candidate saccades occurring between 100 ms and 800 ms after a next location was highlighted on the screen. The first saccade where the sign of the HEOG signal matched the direction of the stimulus-to-stimulus transition was selected as the saccade of interest. If no match was found in the HEOG signal, we looked for saccades in the VEOG with a sign that matched the vertical saccade jump. In 10% of the data no matching saccade was found. The EEG and EOG data was split into segments starting from the point of the highest saccade speed to 1 s after, and baselined using the first 100 ms of the epoch. EEG epochs with extremely high variance (standard deviation exceeding 50 times the standard deviation) were discarded as outliers.

#### Eye Tracking Data

One participant was excluded from the eye data analyses, because of technical difficulties with the eye tracking hardware during this measurement. After the measurement, the fixation locations were recalibrated using the 15 displayed stimulus positions to obtain higher gaze localization accuracy. Fixations were considered to be on the stimulus when the fixation position was within a radius of 150 pixels (4.4 cm or 6.4°) from the center of the current stimulus location. Fixation duration was determined as the time that eye fixation was on the stimulus. Only valid samples in a window starting at stimulus onset until 2 s after were taken into account. Pupil size was determined as the mean of the pupil size values over these same samples.

#### Classification

For classification, we used linear SVM models that were trained to distinguish between either targets vs. non-targets (for each of the load conditions) or hits vs. misses (in the high load condition) using 5-fold cross validation. Classification was performed using the Donders machine learning toolbox developed by van Gerven et al. (2013) and implemented in the FieldTrip open source Matlab toolbox (Oostenveld et al., 2011). The features were standardized to have mean 0 and standard deviation 1 on the basis of data from the training set. Included features were EEG voltages over a time interval of 250–1000 ms after peak velocity of the stimulus saccade, in which all EEG electrodes were included. In order to examine potential information from EOG leaking into EEG, we also used EOG voltages over the same time interval as features. Different models were trained using different combinations of EEG (i.e., SRP) features, EOG features, fixation duration and pupil size. Classification was performed separately for each participant and each load condition. Random selections of non-targets and hits were used in the training sets in order to match the numbers of available target and miss epochs to ensure balanced training of the model. For each participant, each load condition, each type of distinction (target vs. non targets and hits vs. misses) and each model we determined whether classification was above chance using a binomial test. An alpha level of 5% was used.

## Results

### Behavioral Performance

Performance on the secondary (math) task was on average 62% correct (SD 17%) indicating that the secondary task was quite difficult and performance varied strongly between subjects. High workload data from both trials with correct and incorrect responses to the math task were included in the analyses. Note that performance on the math task is no direct indicator of workload. Performance could be high because a participant tried hard (high load) or, for that participant, the sum was easy (low load). Conversely, low performance could be caused by lack of trying (low load) or because the participant simply did not manage, despite trying hard (high load). There was no evidence for participants choosing to focus on the one rather than the other as indicated by the lack of (negative) correlation between participants’ performance on the math task and performance on the monitoring task (Pearson correlation: *r* = 0.25, *p* = 0.27).

**Figure 3:**
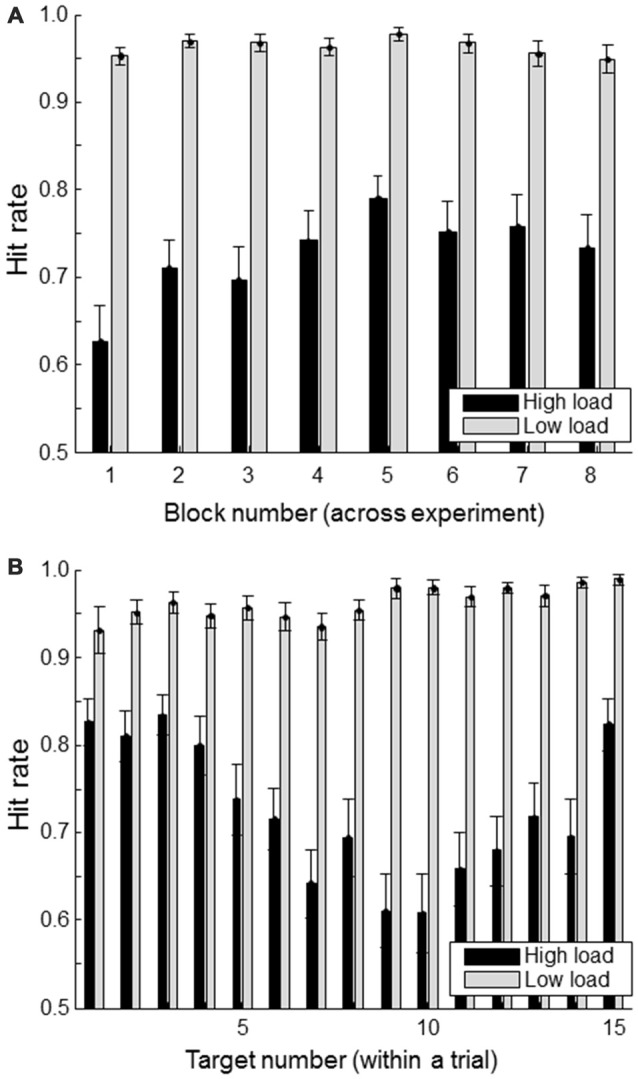
**Hit rate (correctly reported targets as a proportion of the total number of presented targets) separately for load condition**. **(A)** shows hit rate for each of the experimental blocks; **(B)** shows hit rate as a function of a target’s position within a trial consisting of a total of 15 targets and non-targets. Error bars indicate standard errors of the mean.

Figure 3A shows the hit rate of the primary task (defined as the proportion of “#FA#” targets whose location was correctly indicated) for successive blocks (of 11 trials each) in the high and low workload conditions. There seems to be some indication of a learning effect in the high load condition with increasing performance up to block 5. The Figure 3B shows the hit rate as a function of when the target was presented within a trial. For the high load condition, it is clear that targets presented at the beginning or the end of a trial are remembered better than the ones in between. This is consistent with primacy and recency effects.

As expected and intended, the hit rate in the high load condition was much lower (average hit rate of 0.73 (SD 0.13) than in the low load condition (average hit rate of 0.96, SD 0.04). Except for one participant who only missed nine targets, all participants missed at least 22 targets in the high load condition, with an average of 72 missed targets (range: [9, 133]). In the low load condition, the average number of missed targets was eight (with a range of [0, 32]). We consider the average number of eight missed targets in the low load condition as too little to do meaningful hit vs. miss comparisons in this condition.

Missed targets can be accompanied by wrongly identified targets (i.e., false alarms), or not (resulting in less targets being reported than being presented). We found that in general, the latter is the case. In the high load condition, the number of false alarms was on average 42 (range of [5, 132]). Given the number of missed targets, this means that participants reported on average 30 targets less than the number of targets that was actually shown (with a range of [−103, 10]). While brain processes will be different when a target is not identified as a target (definitely leading to not report the target at all) compared to when a target has been identified but not properly encoded (which, depending on the reporting strategy of the participant, could lead to not reporting it at all or to an accompanying false alarm), we do not distinguish between not reporting a target at all and indicating a wrong location in this study. Given the design of our experiment, and the results described in the following, we take both to mean that the target has not been properly encoded. However, it is important to keep in mind that treating these results as the same may not be appropriate under other circumstances.

### Saccade Latencies

Table 1 shows the saccade latencies, defined as the peak velocity of the stimulus saccade relative to stimulus onset. Relative to this point SRP onsets were determined. Latencies toward targets that are missed are longer than toward hit targets (*t*_(19)_ = 2.18, *p* = 0.04 for the low load condition; *t*_(19)_ = 4.85, *p* < 0.01 for high load). Saccade latencies toward targets are shorter than toward non-targets. Although the difference is small (8 ms in both low and high load conditions), it is statistically significant (respectively (*t*_(19)_ = 2.83, *p* = 0.01 for low load; *t*_(19)_ = 2.12, *p* = 0.05 for high load). We think that this effect is mediated by a longer fixation duration for targets than non-targets (see “Eye—Fixation Duration” Section). The object fixated prior to a target is more likely to have been a non-target (that does not keep the eyes linger relatively long and results in a short saccade latency for the next object) compared to the object fixated prior to non-target.

**Table 1:** **Means and standard deviations of saccade latency (time of peak velocity of the stimulus saccade relative to stimulus onset), separately for each low and high load condition, targets, non-targets, hits and misses**.

	Low load		High load	
	mean	SD	mean	SD
Target	157	40	221	52
Non-target	165	45	229	53
Hit	154	38	199	43
Miss	267	245	284	97

*The gray font of the hits and misses in the low load condition signifies that because of few misses in the low load condition, the hit trials are about the same as the target trials and the misses are represented by few data points*.

### EEG—SRP

**Figure 4:**
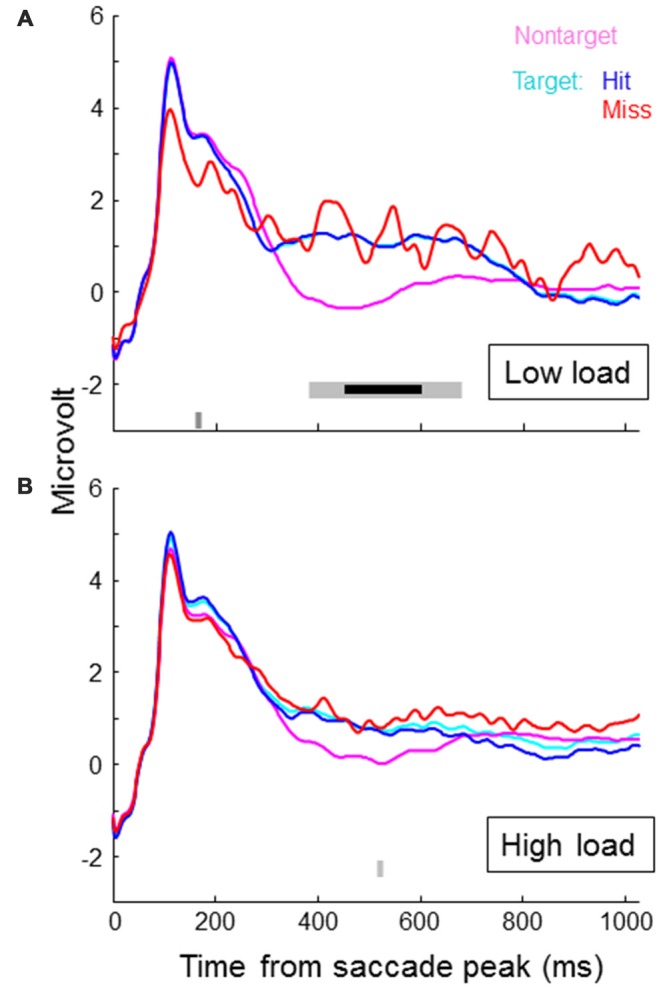
**Saccade related potential (SRP) traces averaged across participants and electrode locations CP1, P3, Pz, PO3, PO4, P4 and CP2, separately for non-targets and targets, where targets are again separated in hits and misses**. Onset of the SRP traces is the time of peak velocity of the saccade towards the (non-) target. **(A)** shows results for the low load condition, the **(B)** for the high load condition. In the lower part of the figures we indicated individual time samples (3.9 ms) that were significantly different for target vs. non-target (light gray) and hit vs. miss (dark gray) as indicated by paired *t-tests*.

Figure 4 shows SRP traces associated with targets, non-targets, hits and misses for each of the two load conditions, averaged across participants and electrode locations around Pz (CP1, P3, Pz, PO3, PO4, P4, CP2). In the lower part of the figures we indicated individual time samples (3.9 ms) that were significantly different for target vs. non-target (light gray) and hit vs. miss (dark gray) as indicated by paired *t*-tests (alpha level of 5%). Around 500 ms, target SRPs are larger than non-target SRPs, which is consistent with a stronger P300 for targets than non-targets. The difference appears stronger in the low load condition—only in this condition, the difference between target and non-target traces reached significance for an uninterrupted interval of almost 300 ms. After correcting for multiple testing (Benjamini and Hochberg, 1995), this interval is reduced to around 150 ms (indicated by the bold, black line in Figure 4). When examining traces for load condition separately, hit and miss traces did not differ for a substantial period of time. When collapsing across load conditions, the higher values for miss traces towards the end of the epoch becomes significant. However, it is clear that at least up to 450 ms, miss traces overlap with hit traces. Miss traces certainly do not lie in between hit and non-target traces.

Exploring average traces for all individual channels revealed no significant differences between target and non-target, and hits and misses in the math condition. For the non-math condition, we found significant effects as reflected in Figure 4 (higher voltages for targets compared to non-targets around 500 ms) for channel CP1, Pz and P4. P7 showed a lower voltage for targets compared to non-targets around 250 ms, and F8 a lower voltage around 450 ms.

### Eye—Fixation Duration

**Figure 5:**
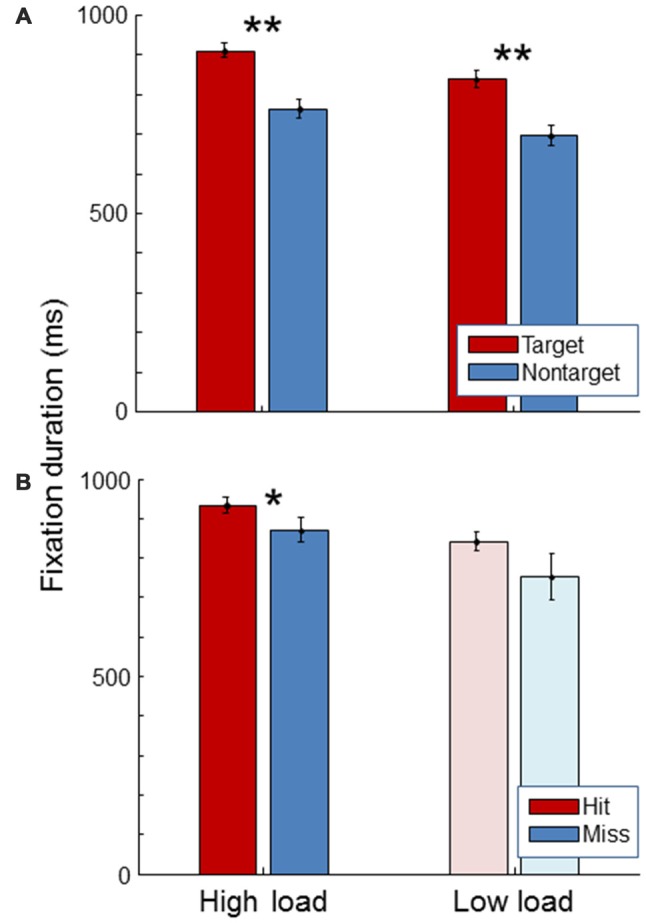
**Average fixation duration for the high load and low load condition, separately for targets and non-targets (A)** and, for the targets, separately for hits and misses **(B)**. The light color of the low load condition bars in the **(B)** signify that these represent little data since there were few misses in the low load condition. Error bars indicate standard errors of the mean. Stars indicate significant differences (**representing *p* < 0.01, *representing *p* < 0.05).

Figure 5 shows average fixation duration. As expected, fixation duration was longer for targets than for non-targets, both in the high and in the low load condition (paired *t*-tests, *t*_(19)_ = 11.47, *p* < 0.01; *t*_(19)_ = 11.43, *p* < 0.01). Fixation duration was longer for hits than for misses in the high load condition (paired *t-test*, *t*_(19)_ = 2.38, *p* = 0.03). The same trend was seen in the low load condition. No significant difference was found in fixation duration between high and low workload conditions (paired *t*-test, *t*_(19)_ = 1.40, *p* = 0.18).

### Eye—Pupil Size

**Figure 6:**
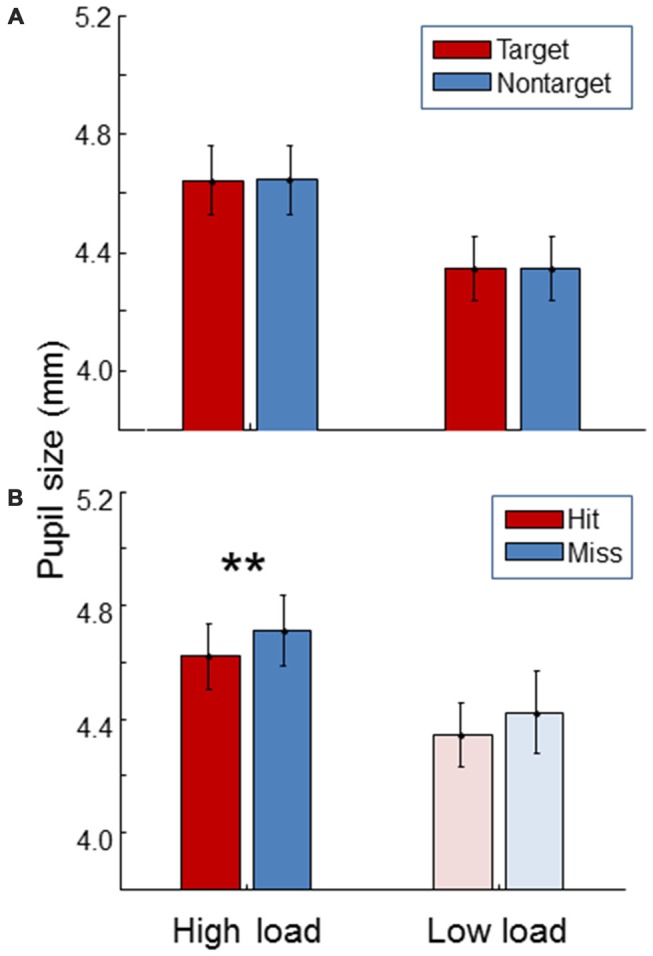
**Average pupil size for the high load and low load condition, separately for targets and non-targets (A)** and, for the targets, separately for hits and misses **(B)**. The light color of the low load condition bars in the **(B)** signify that these represent little data since there were few misses in the low load condition. Error bars indicate standard errors of the mean. Stars indicate a significant difference (*p* < 0.01) between hits and misses.

Figure 6 shows pupil size. For both high and low load conditions, pupil size was the same between targets and non-targets (paired *t*-test, *t*_(19)_ = 0.54, *p* = 0.59; *t*_(19)_ = 0.12, *p* = 0.91). As expected, pupil size was significantly larger during the high load condition compared to the low load condition (paired *t*-test, *t*_(19)_ = 12.5, *p* < 0.01). Additionally, pupil size was found to be larger for misses than for hits in the high workload condition (paired *t*-test, *t*_(19)_ = 4.25, *p* < 0.01). The same trend was found in the low load condition.

### Single Trial Analysis

**Figure 7:**
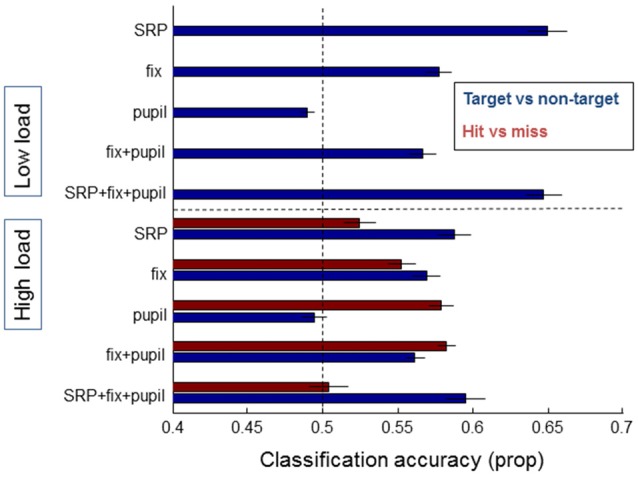
**Classification results of models averaged over individual participants that distinguish between target and non-target fixations (blue bars) and hits and misses (red bars), separately for data from the low load condition (upper half of the figure—no results for hit and miss due to few misses) and the high load condition (lower half of the figure)**. On the left the features that the models were based on are indicated: SRP, fixation duration, pupil size, combined eye features, and a combination of all features. Error bars indicate standard errors of the mean.

Figure 7 shows the classification accuracy between targets and non-targets (blue bars) separately for the low and high load conditions, and hits and misses (red bars) for the high load condition. As expected from the average SRPs as presented above and from previous studies, single fixation classification was possible for target vs. non-targets based on SRP in the low load condition (on average 65% correct, with performance significantly above chance for 13 out of 21 participants according to binomial tests). For the high load condition average classification performance on the basis of SRP reaches 59% correct (7 out of 21 participants above chance). For both load conditions, classification accuracy between target and non-targets is highest when SRP features are used. Adding fixation duration and pupil size does not (substantially) improve performance.

Another picture emerges for the distinction between hits and misses—which we could only meaningfully examine for the high load condition due to the very small number of misses in the low load condition. Classification based on SRPs is around chance level (52% correct), while classification based on pupil size results in an average single fixation classification accuracy of 58%. Adding fixation duration does not (substantially) improve performance; adding SRP rather decreases performance. Distinguishing between hits and misses did not reach above chance performance for single subjects (note that the power of the binomial tests is much weaker for hits vs. misses compared to targets vs. non-targets where more data was available).

**Figure 8:**
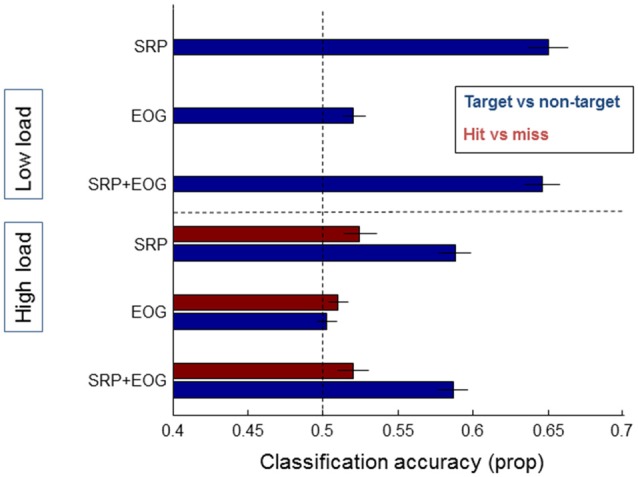
**Classification results of models averaged over individual participants that distinguish between target and non-target fixations (blue bars) and hits and misses (red bars), separately for data from the low load condition (upper half of the figure—no results for hit and miss due to few misses) and the high load condition (lower half of the figure)**. On the left the features that the models were based on are indicated: SRP, EOG, and a combination of SRP and EOG features. Error bars indicate standard errors of the mean.

Figure 8 shows results for classification based on EOG features, and EOG and SRP features combined, with the result of the SRP based models as a comparison. When using EOG, classification accuracy between either targets and non-targets or hits and misses does not rise over 52% and adding EOG to SRP does not improve classification.

## Discussion

We investigated a structured search task where fixated targets that observers can easily identify are relatively likely to remain unreported due to a concurrent secondary task that is expected to interfere with working memory. We found that in such a case, SRPs differ between targets and non-targets (consistent with a target eliciting a P300), but not between hit targets and missed targets. Fixation duration was longer for targets than non-targets as expected, and also for hits compared to misses. Pupil size did not differ between targets and non-targets, but was larger for misses than for hits. In sum, EEG features appeared more suitable to distinguish targets from non-targets while eye features (especially pupil size) were suitable to distinguish fixated targets that were subsequently reported from those that were not. These results were also reflected in single trial classification analyses.

We interpret our findings as reflecting distinct underlying cognitive processes as discussed further below.

### SRP P300

Differences between target and non-target SRPs are as expected with a higher amplitude P300 for targets compared to non-targets. This difference is smaller between the average target and non-target SRP traces in the high compared to the low load condition. Previous studies also found the P300 to be less pronounced under high load conditions (Allison and Polich, 2008; Gherri and Eimer, 2011; Pratt et al., 2011). Also for ERPs following fixations, smaller target P300s during high workload compared to low workload have been reported before Ries et al. (2016). This reducing effect of workload on the P300 is consistent with less attentional resources being available for the target detection task.

While the P300 reflects attentional processes, this did not translate to larger P300s for hit compared to missed targets. Clearly, SRPs associated with missed targets more closely resembled hit target rather than non-target SRPs. This suggests that, as we intended, there is no problem in target identification and that the amount of attention allocated to the fixated object around the time of fixation is not critical for its encoding in memory.

### Fixation Duration

As expected, fixation duration is longer for targets than for non-targets. We also found it to be longer for hits than for misses. A short duration of a fixation being associated with misses could in principle be caused by participants moving the eyes from the target to the next stimulus too quickly, or because participants lingered relatively long on the stimulus that was fixated before the target, which causes a late arrival on the target and leaves less time for target fixation. Comparing the differences in hit-miss saccade latency to the difference in hit-miss fixation duration indicates that misses are mostly due to arriving late rather than leaving too early.

Note that in the present structured search task, potentially relevant information appeared every second, encouraging rather long, and relatively invariable fixation durations. Thus, in different types of search tasks fixation duration effects can be expected to be stronger.

Also note that the rather strong association between fixation duration and hits and misses could potentially have led to confounding effects in the SRPs, i.e., differences between hit and miss SRPs that cannot be attributed to memory or attentional processes but are e.g., due to visual processes caused by systematic timing differences in the change of stimulus appearance. In this study, we did not find any significant difference between hit and miss SRPs, but this should be kept in mind for similar future studies.

### Pupil Size

Pupil size is larger in the high load compared to the low load condition. An increased pupil size with an increase in memory load or workload is a robust finding in the literature (Kahneman and Beatty, 1966; Beatty, 1982; Hogervorst et al., 2014). We also observed the hypothesized effect of larger pupil size during missed targets, consistent with a momentary high workload being the cause of the miss. Such momentary increase in cognitive workload could have been caused by a difficult (part in the) math trial, or by having to store yet another target. Also, momentary fluctuations could be caused by participants sometimes giving up on the math task half way during a trial. Our data do not reflect differences in phasic pupil size responses—there was no significant difference in pupil size between targets and non-targets as determined by the median size over the fixation interval. While our dependent measure of pupil size was not optimal to capture differences in pupil dilation that start to diverge at longer latencies and reach their maximum difference at about 1.5 s post fixation (Hong et al., 2014; Jangraw et al., 2014), averaged pupil size traces in our data did not show the expected differential pattern for targets and non-targets. At present, we do not know why this difference was not observed. The average pupil size was quite large (around 4.5 mm diameter) which may have played a role in attenuating the phasic response.

In sum, SRP and eye features provide complementary information in the search task under study. Saccade-related P300s signify that a target has been detected but not that it has been encoded for successful recall. For that, fixation duration and pupil size are informative, likely because they respectively reflect time taken to store the target and variations in workload associated with the secondary task.

### Application

Determining whether an observer is looking at a target (i.e., at an object that is of interest) using SRPs and eye related features may be useful in two general areas. If it is not known what visual information is important (in a particular situation, or for a particular person) such features provide a means to examine what is of interest to an observer without requiring conscious behavioral responses. If it is known what information is important, brain and eye signals can be used to judge whether this important information is properly recognized as being important. If we know whether an observer is gazing at relevant information (as judged from the brain and eye signals, or as *a priori* knowledge of the task), determining whether this information is going to be remembered could be used for deciding whether or not to present the information again or in another way. Target recognition and encoding indicators may also be used as indicators of (momentarily) suboptimal performance so that the system can advise the human observer or operator to take break. The interplay between user state detection and responses to individual targets may be used in higher level classification strategies. If observers are detected to be in a high memory load situation (because of a large pupil size and EEG features), a model that classifies fixated targets (as detected through SRP) into hits and misses may be activated.

In addition to physiological or eye-based sources of information, task- and context-related information can be used directly in models for state monitoring and behavior prediction. For instance, knowledge of the presence of an additional or difficult task will add to the likelihood of misses, and indicate that a classifier distinguishing between hits and misses should be put to work. In some cases, knowledge of the task may strongly influence the interpretation of eye and brain signals that occur while searching and storing visual information. As mentioned before, low workload as may be indicated by a small pupil size, can be associated with misses in cases that a target is difficult to identify and there are no other tasks (the situation as in Dias et al., 2013) or it can be associated with hits in cases that a target is easy to identify and there is a concurrent task (current study). Also, the type of search task influences which physiological or eye based features can be expected to be informative. When there is no time pressure, fixation duration is expected to be more informative compared to when there is.

It has to be realized that the certainty with which it is possible to determine whether an observer is looking at a target or whether an observer is going to miss a target at the individual fixation level can never achieve perfect accuracy. The highest fixation classification accuracy as found in the current study is 65% and associated with distinguishing targets from non-targets in the low load condition. While it is obviously difficult to compare classification performance across studies, a classification accuracy of around 65% (with a 50% chance level) has also been found in other studies distinguishing targets from non-targets using SRPs (Brouwer et al., 2013: 62%; Wenzel et al., 2016: 67%; Kaunitz et al., 2014: 63%). SRP studies that report a relatively good performance (Touryan et al., 2016: up to around 82%; Ušćumlić and Blankertz, 2016: a mean AUC of almost 80%) employed methods of subtracting out eye movement artifacts. Brouwer et al. (2014) found a decrease of the Equal Error Rate for distinguishing between target and non-target SRPs from 40% to 31% when removing eye artifacts. Amongst a range of possible techniques to boost classification performance, removing eye movement components from SRP traces seems to be an important one.

For real applications it is also important to consider the context and circumstances where uncertain information about “targetness” or memory encoding, based on eye and brain signals, are expected to add to other available or easy-to-retrieve information such as behavioral data (Brouwer et al., 2015). Preferably, this would be in an environment with minimal noise affecting the brain and eye signals, but also situations where the signal is strong—e.g., low workload scenarios are beneficial when exploiting the P300 signal to distinguish between targets and non-targets (current study, Thurlings et al., 2013; Ries et al., 2016). An example that may capture these elements is robust image triage. If a subset of rapidly presented images is identified for careful review and inspection (e.g., x-ray images of luggage), it would be prudent to include images with a relatively high risk of (missed) targets.

## Author Contributions

All authors contributed to the conception of the study, analysis and writing.

## Funding

This research was sponsored by the US Army Research Laboratory and was accomplished under Contract Number W911NF-10-D-0002.

## Conflict of Interest Statement

The authors declare that the research was conducted in the absence of any commercial or financial relationships that could be construed as a potential conflict of interest.
